# Isolation and Quantification of Plasma Cell-Free DNA Using Different Manual and Automated Methods

**DOI:** 10.3390/diagnostics12102550

**Published:** 2022-10-20

**Authors:** Eleni Polatoglou, Zsuzsanna Mayer, Vida Ungerer, Abel J. Bronkhorst, Stefan Holdenrieder

**Affiliations:** Munich Biomarker Research Center, Institute of Laboratory Medicine, German Heart Centre, Technical University Munich, Lazarettstraße 36, D-80636 Munich, Germany

**Keywords:** liquid biopsy, cell-free DNA, plasma, cell-free DNA isolation

## Abstract

Plasma cell-free DNA (cfDNA) originates from various tissues and cell types and can enable minimally invasive diagnosis, treatment and monitoring of cancer and other diseases. Proper extraction of cfDNA is critical to obtain optimal yields and purity. The goal of this study was to compare the performance of six commercial cfDNA kits to extract pure, high-quality cfDNA from human plasma samples and evaluate the quantity and size profiles of cfDNA extracts—among them, two spin-column based, three magnetic bead-based and two automatic magnetic bead-based methods. Significant differences were observed in the yield of DNA among the different extraction kits (up to 4.3 times), as measured by the Qubit Fluorometer and Bioanalyzer. All kits isolated mostly small fragments corresponding to mono-nucleosomal sizes. The highest yield and reproducibility were obtained by the manual QIAamp Circulating Nucleic Acid Kit and automated MagNA Pure Total NA Isolation Kit. The results highlight the importance of standardizing preanalytical conditions depending on the requirements of the downstream applications.

## 1. Introduction

Cell-free DNA (cfDNA) is a minimally invasive and real-time biomarker for the early detection, identification and monitoring of various diseases. Liquid biopsy has been investigated as a minimally invasive technique that can avoid the inherent shortcomings of tissue biopsy, such as sampling bias, tissue heterogeneity and difficulty in repetitive sample extraction [1,2]. However, due to the great variability and relatively low abundance (average 10–30 ng/mL, range 1.8–44 ng/mL) [3,4] of cfDNA in circulation and the high degree of fragmentation, it remains a highly challenging analyte.

Preanalytical factors can significantly affect the quality and quantity of cfDNA and have to be investigated thoroughly [5]. Such factors include the choice of matrix (plasma or serum), the sample collection tubes and processing (centrifugation regime), storage, thawing conditions (temperature and freeze–thaw cycles), DNA isolation method, storage of isolated DNA, method of quantification and the intended downstream analysis [6,7,8]. General requirements for efficient isolation techniques include fast, robust, simple and automatable methods that extract cfDNA with satisfactory purity and yield. This is crucial to ensure reliable results in downstream applications such as NGS or PCR. However, the method of choice also depends on the number and volume of samples.

There is a variety of commercially available DNA isolation methods based on different binding chemistries. Each of these chemistries can influence the efficiency and purity of the isolation, and each has a characteristic binding capacity. Ethanol precipitation, anion-exchange resin (coupled with ethanol precipitation), silica gel membrane binding and magnetic silica particle binding technologies can be used. Out of these, magnetic particle-based methods are cheaper, faster and easier to upscale and automate, while membrane binding methods can provide higher yields. The method of choice depends on the number of samples and their volume, required output, purity and downstream applications. Most commercial kits favor the isolation of DNA fragments from 50 to 800 bp, which encompasses the generally reported size of cfDNA (130–170 bp) [9]. However, it is increasingly recognized that the view of mono-nucleosomes as the most important fraction of cfDNA is biased. Recently, smaller fragment sizes have been shown to be of clinical interest [10,11,12], and there is a growing body of evidence that a significant portion of longer cfDNA fragments may not represent contaminants form lysed peripheral blood cells, such as extrachromosomal circular DNA [13,14]. Long cfDNA fragments (larger than 1 kb) from hepatocellular carcinoma patients have recently been shown to contain lower methylation levels than those from non-cancer patients and carry characteristic methylation patterns, opening new possibilities for cancer liquid biopsy [15].

The goal of this study was to compare six commercial cfDNA kits to extract pure, high-quality cfDNA from human plasma samples and evaluate the quantity and size profiles of the obtained extracts.

## 2. Materials and Methods

### 2.1. Plasma Samples

First, 11 mL plasma samples were collected in Sarstedt S-Monovettes 9 mL K3E (1,6 mg K3 EDTA/mL blood; Sarstedt Diagnostics GmbH; Nürmbrecht; Germany) from ten healthy individuals. These samples were collected as part of the quality control for biobanked blood samples. Blood samples were taken from patients of the Department of Cardiology during routine venipuncture or from healthy individuals after informed consent for blood collection for the Cardiovascular Biobank of the German Heart Centre Munich was obtained. Blood collection for biobanking was approved by the Ethics Commission of the Technical University Munich (Nr. 5943/13; 16.10.2013).

### 2.2. Sample Processing

After blood drawing, samples were kept at room temperature and were processed within 60 min. First, they were centrifuged at 1600× *g* for 10 min at 20 °C. Then, the upper layer was transferred into a 15 mL Falcon tube (BD, New Jersey, United States) and centrifuged again at 6000× *g* for 10 min at 20 °C. The plasma was aliquoted into 1.5 mL Eppendorf Safe-Lock tubes (Eppendorf, Hamburg, Germany) and stored at −80 °C within 30 min of the second centrifugation. Before further processing, all samples were thawed at room temperature. After isolation, all DNA samples were stored in 1.5 mL DNA-LoBind tubes (Eppendorf) at −20 °C.

### 2.3. cfDNA Isolation

Five manual magnetic bead or spin column methods and one automated method were used in a direct comparison. The isolation kits were: QIAamp Circulating Nucleic Acid Kit (QiaM, 55114 Qiagen GmbH, Hilden, Germany), NucleoSpin Plasma XS (Macherey-Nagel 740900.50, high-sensitivity protocol—MNaS, Macherey-Nagel GmbH, Düren, Germany), QIAmp MinElute ccfDNA Mini Kit (QiaS, 55204, Qiagen GmbH, Hilden, Germany), cfPure Cell-Free DNA Extraction Kit (BChM, K5011610-BC, BioChain Inc., Newark, CA, USA), MagMAX Cell-Free DNA Isolation Kit (TFiM, A29319, Thermo Fisher Scientific, Waltham, MA, USA) and the automated method MagNA Pure 24 Total NA Isolation Kit (RocA, 07658036001, Roche Diagnostics GmbH, Penzberg, Germany), using the cfNA ss 2000 protocol on the MagNA Pure 24 System (Roche Diagnostics). All kits and their code names for this study are summarized in Table 1. Isolations were performed in duplicate following the instructions provided in the manual of each kit. Moreover, 1 mL of plasma was used as input, with the exception of the MNaS kit, wherein 240 µL was used. For RocA, 1 mL of PBS was added to 1 mL of plasma to obtain the required 2 mL. The elution volume for QiaM and QiaS was 50 µL, for BChM and TFiM 30 µL, for MNaS 12 µL and for RocA 100 µL.

### 2.4. cfDNA Quantification and DNA Sizing

The concentration of the isolated cfDNA was analyzed in duplicate by fluorometric quantification using the Qubit Fluorometer 3.0 (Thermo Fisher, Waltham, MA, USA) and the dsDNA HS Assay (quantification range: 10 pg/µL–100 ng/µL; Thermo Fisher). For four of the donors, we determined the representative quantity and fragment size profiles of cfDNA using a 2100 Bioanalyzer (Agilent Inc., Santa Clara, CA, USA) with the Agilent High-Sensitivity DNA Kit.

### 2.5. Statistics

Statistical analysis of the data was performed using the GraphPad Prism software version 8 and Excel. Differences between group means were calculated using one-way analysis of variance (ANOVA), followed by pairwise comparison using a post-hoc Tukey test. Differences were considered statistically significant if the *p* values were smaller than 5% (*p* < 0.05).

## 3. Results and Discussion

**Figure 1:**
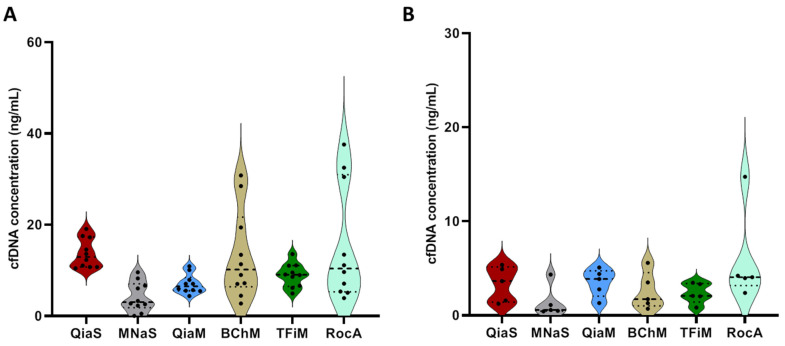
Comparison of cell-free DNA (cfDNA) yields delivered by different DNA extraction kits. CfDNA was isolated from the plasma of 10 healthy donors using different extraction kits and then quantified by (**A**) the Qubit HS DNA kit and (**B**) the Agilent HS DNA Kit on the Bioanalyzer. Quantitative measurements of cfDNA are expressed as the total mass of cfDNA (ng) present in 1 mL of plasma.

CfDNA is a source of clinical biomarkers in blood with applications ranging from cancer detection and monitoring to prenatal diagnostics. However, with concentrations ranging from a few ng/mL to several thousand ng/mL [3,16] and various fragment sizes, cfDNA is challenging to analyze. In this study, we compared six different extraction kits, including two spin column-based methods and four magnetic bead-based methods, to one that is automated. We isolated cfDNA from 1 mL plasma for each healthy volunteer (for Nucleospin Plasma XS (MNaS), only 240 µL plasma was used) and the yield and fragment sizes were assessed with the Qubit Fluorometer and Bioanalyzer (BA), respectively. Based on the Qubit and BA measurements, all methods were able to recover cfDNA from all plasma samples (Figure 1).

**Figure 2:**
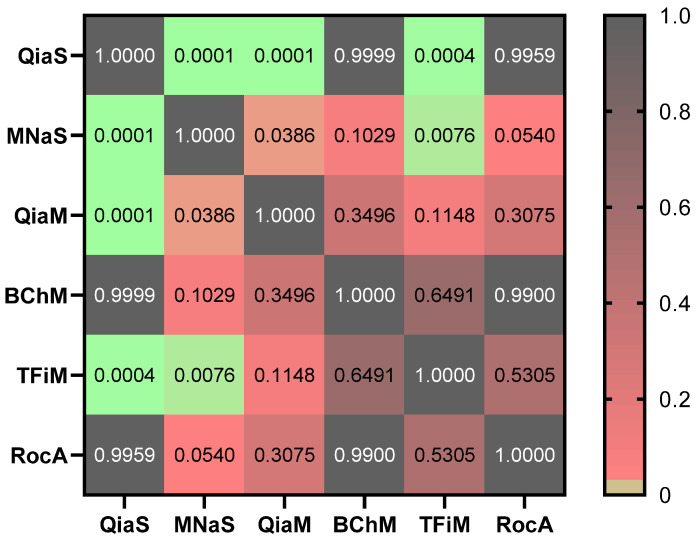
A heat map representation of the post-hoc multiple comparison of cfDNA recovery isolated using different extraction kits and measured with the Qubit HS DNA assay. Significant differences (*p* < 0.05) are shown in green to orange.

Overall, QiaS showed significantly greater recovery in comparison to MNaS (*p* = 0.0001), QiaM (*p* < 0.0001) or TFiM (*p* = 0.0004) when assessed by Qubit. No statistically significant difference was observed between QiaS and BChM or RocA. In addition, MNaS produced significantly lower yields compared to QiaM (*p* = 0.0386), TFiM (*p* = 0.0076) or QiaS. No other statistically significant difference was observed (Figure 2). Although spin column-based methods are typically more costly and time-consuming than magnetic approaches, they typically produce higher yields. In our study, QiaS resulted in the highest recovery, which is consistent with a number of comparative studies [5,17,18,19,20,21,22].

The method of quantification can also significantly affect the measured quantity of DNA. For example, Maas et al. 2021 compared the Qubit, PicoGreen assay, BA and TapeStation and found that the Qubit, BA and TapeStation provide excellent estimations of the sample concentrations, especially within the ranges specified by the manufacturer [23]. A few years prior, though, Solassol et al. demonstrated that the Qubit was superior to the TapeStation, especially for estimating higher concentrations [24]. In our analysis, cfDNA concentrations were lower when quantified by the BA as compared with the Qubit, and there were only minor significant differences between the various extraction methods when concentrations were assessed by the BA. MNaS resulted in significantly lower concentrations than QiaM (*p* = 0.0052) and TFiM (*p* = 0.03496). Additionally, BChM resulted in significantly lower concentrations than QiaM (*p* = 0.0335) and TFiM (*p* = 0.0377). This may be related to the fact that TFiM consistently isolated longer DNA fragments (>1 kb), which are excluded from the cfDNA estimation in the BA. A similar finding was reported by Cédile et al. in 2021, when they observed an underestimation of cfDNA plasma concentrations by BA (2.86–14.12 ng/mL) as compared with Qubit measurements (7.5–23.31 ng/mL) [25].

To estimate the reproducibility of each extraction method, the coefficient of variance for each sample was calculated and each CV% value was used to calculate the median CV% of each method, thereby avoiding a direct influence of sample variations. Median CV% and ranges are shown in Table 2. The smallest variation was found for QiaS (11.8%), followed by RocA (16.0%), which agrees with the observations of van Ginkel et al., 2017 [26]. The two methods also displayed comparable yields of cfDNA. BChM was found to have the lowest reproducibility, with a CV% of 34.6%, which is reported to show excellent reproducibility in the cell culture context [22]. Here, it is noteworthy that, in the context of plasma, there is often higher variability between replicate isolations, and although CV% values are not commonly reported (e.g., when each sample is isolated only once), we often encounter large standard deviations, especially for low amounts of cfDNA [19,23]. Variability seems to be lower for spike-in controls [17].

**Figure 3:**
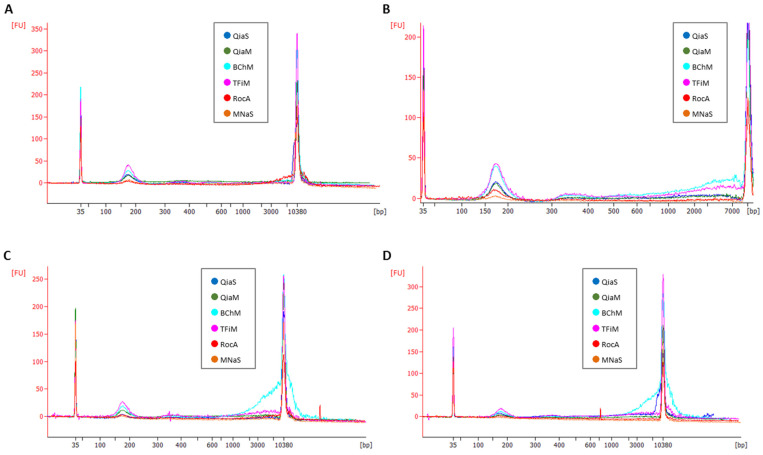
Cell-free DNA (cfDNA) size profiles yielded by different extraction kits. (**A**–**D**) Representative electropherograms from 4 of the 10 donors, as measured with the Agilent HS DNA Kit on the 2100 Bioanalyzer. The curves in these figures are not representative of the total cfDNA yield by the respective kits, as different elution volumes were used for the different extraction kits (100 µL for RocA, 50 µL for QiaS and QiaM and 30 µL for TFiM and BChM).

To evaluate the size distribution of the isolated cfDNA fragments extracted using the different kits, four of the ten isolated samples (samples from 4 of the 10 donors) were run on the Bioanalyzer using the Agilent High-Sensitivity DNA Kit (Figure 3).

**Figure 4:**
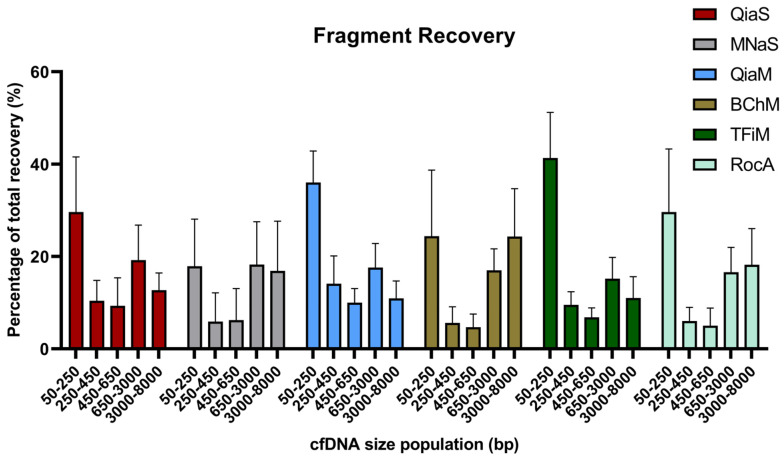
Size bias of different cell-free DNA (cfDNA) extraction kits. The cfDNA size profiles delivered by the six different DNA extraction kits, as measured by the Agilent HS DNA Kit on the 2100 Bioanalyzer, were analyzed to determine the relative proportions of different size populations recovered by each kit. Each bar represents that average of four donor samples. Error bars indicate standard deviation.

The on-board software of the BA was then used to calculate the relative proportions of different cfDNA size populations recovered by each method (Figure 4). Taken together, these size analyses showed that all kits recovered a high proportion of mono-nucleosomes, while all kits also recovered a small percentage of larger cfDNA fragments, where the BChM kit delivered the highest relative proportion of longer cfDNA fragments. The relative proportion of mono-nucleosomes in the eluates appears to be lower than what is commonly reported in plasma. This is most likely due to the low amounts of DNA extracted, which leads to an overestimation of the longer fragments by the BA as the background noise becomes higher. Thereby, all kits resulted in similar percentages of mono-nucleosomes, without significant differences, as measured with the dsDNA High-Sensitivity Kit on the Bioanalyzer. The median percentage for MNaS was 21%; 22% for BChM and RocA, 26.5% for QiaS, 34% for TFiM and 34.5% for QiaM. The same effect was seen in Maas et al. 2022, when they assessed the sensitivity and reproducibility of the BA and other methods in low concentration ranges [23]. Therefore, in our case, the Qubit is the preferred method for quantification, while the BA still provides valuable information about shorter fragment sizes, such as those of mono- and di-nucleosomes.

## 4. Conclusions

Based on our results, we selected QiaS as the kit most suited to our needs. This kit produces cfDNA at a desirable concentration for downstream applications such as use with PCR and DNA sequencing. RocA delivers comparable cfDNA yields and it has some benefits over manual methods (better standardization), and it may be more suitable for high-throughput laboratories. BChM can result in higher yields of longer DNA fragments. The choice of kit will depend on the individual needs of the user. In the clinical setting, it is important to obtain the highest DNA quantity from as little sample volume as possible, in order to enable multiple tests even with precious and spare samples. In a future comparison, it would be interesting to see how different isolation methods show bias towards the capturing of cfDNA fragments that are both shorter and longer than mono-nucleosomes in the context of human plasma.

## Figures and Tables

**Table 1:** CfDNA extraction kits compared in this study.

Product	Code	Type	Can Be Automated	Isolation Volume (mL)	Elution Volume (µL)
QIAamp Circulating Nucleic Acid Kit (Qiagen)	QiaS	spin column (vacuum manifold)	no	1	50
NucleoSpin Plasma XS (Macherey-Nagel)	MNaS	spin column	no	<0.24	5–30
QIAmp MinElute ccfDNA Mini Kit (Qiagen)	QiaM	magnetic beads	yes	1–4	20–80
cfPure Cell-Free DNA Extraction Kit (BioChain)	BChM	magnetic beads	yes	1–10	15–50
MagMAX Cell-Free DNA Isolation Kit (Thermo Fisher Scientific)	TFiM	magnetic beads	yes	0.5–10	15–50
MagNA Pure 24 Total NA Isolation Kit (Roche)	RocA	magnetic beads (automated)	-	2	50/100

**Table 2:** Reproducibility of cfDNA recovery of different kits.

Kit	Code	Median Conc. (ng/mL)	CV% Range	Median CV%
QIAamp Circulating Nucleic Acid Kit	QiaS	13.0	8.3–21.9	11.8
NucleoSpin Plasma XS	MNaS	3.0	3.1–64.8	16.2
QIAmp MinElute ccfDNA Mini Kit	QiaM	6.5	5.3–29.6	20.0
cfPure Cell-Free DNA Extraction Kit	BChM	10.2	3.5–52.4	34.6
MagMAX Cell-Free DNA Isolation Kit	TFiM	9.0	2.9–75.2	21.5
MagNA Pure 24 Total NA Isolation Kit	RocA	10.4	3.2–44.4	16.0

## Data Availability

Not applicable.
